# Lipidomics Conquers a Niche, Consolidates Growth

**DOI:** 10.3390/ijms20133188

**Published:** 2019-06-29

**Authors:** David Touboul, Mario Ollero

**Affiliations:** 1 Institut de Chimie des Substances Naturelles, CNRS UPR 2301, Univ. Paris-Sud, Université Paris-Saclay, 8 Avenue de la Terrasse, 91198 Gif-sur-Yvette, France; 2 Institut Mondor de Recherche Biomédicale, INSERM, U955 EQ21, 8, rue du Général Sarrail, 94010 Créteil, France; 3 Université Paris Est Créteil, 61, avenue du Général de Gaulle, 94010 Créteil, France

Sixteen years after the first published article in which the term “lipidomics” was stated [1], one of the latecomers to the omics revolution has consolidated its position in the evolution of analytical approaches in experimental biology and has conquered a specific niche in science. More than 3000 publications since the pioneering work by Han and colls. (colleagues) confirm that widespread lipidomics research has become a reality, even if constituting a modest production as compared to that of proteomics and genomics. As shown in Figure 1, the number of articles published on genomics and proteomics seems to have plateaued since 2017, according to PubMed. Meanwhile, the “minor” omics disciplines (transcriptomics, metabolomics, glycomics, and lipidomics) continue to grow exponentially (Figure 1a). Lipidomics has undergone an explosion of publications since 2013 and continues to experience a sustained and impressive growth (Figure 1b). Most strikingly, since the publication of the last Special Issue on “Bioactive Lipids and Lipidomics”, in 2015, more articles have been published on lipidomics than in the preceding twelve years (2003–2012) (Figure 1c).

The current special issue provides an instantaneous picture of the situation and witnesses the place of lipidomics in terms of technological advances and fields of application, and hints about the directions research may follow in the near future.

## 1. Technology

As a technology-driven discipline, the evolution of lipidomics is directly linked to that of the associated technologies for the separation, detection, and identification of compounds. While analytical approaches are consolidated and can be globally applicable to most omics, the specific technological development presently resides in data processing and molecular networking analysis. 

A particular challenge is still the identification and characterization of molecules. The combination of mass spectrometry data with other technologies, such as optical rotation analysis and NMR [2], provides functional and structural insight. Differential scanning calorimetry is utilized in the quality control of lipid biomaterials [3].

The integration of different omics analyses has been one of the main challenges of global analysis strategies. Stuani and colls. successfully combined proteomics and lipidomics to address fatty acid metabolism in combination with stable isotope labeling [4]. 

Supercritical fluid chromatography, addressed in our 2015 special issue [5], is represented in this new edition by a study in which it is applied to glycosphingolipid analysis [6]. In the same study, this novel approach is combined with isotope labelling and high-performance thin layer chromatography to scrutinize metabolic flux, showing the relevance of classic strategies. This is also the case of GC-MS for fatty acid profiling (4). An alternative to the latter is the selective ion monitoring-tandem mass spectrometry (SIM-MS/MS), used in this case for fatty acid analysis in extracellular vesicles [7] and tissues [3].

Lipid imaging remains a promising field, and a cluster TOF-SIMS strategy is presented by Abbas and colls. to detect lipid ions at the kidney glomerular scale [8].

As emerging technologies allow for increasing sensitivity of measurement, studies have to deal with a larger number of variables, thus requiring a profound network analysis. This is being performed with the help of software tools like MZmine [9], IDEOM [10], or online open source platforms, like Cytoscape (https://cytoscape.org/). Databases like the one provided by the Dictionary of Natural Products are of great help.

## 2. Applications

The expansion of lipidomics leads the discipline to conquering new areas. This includes non-global studies targeting novel bioactive lipids.

The analysis of natural molecules is gaining importance. The need for new perspectives in therapeutics or plague control is directly linked to technological advances. Thorough analyses of new natural sources of bioactive molecules, such as endophyte products (as published by Barthélemy and colls [9]), are highly valuable. The antimicrobial and insecticidal effects of lipoamino acids from entomopathogens have also been characterized by Touré and colls [2]. Abnormal lipid metabolism due to a frequent mutation in acute myeloid leukemia cells has been revealed by Stuani and colls [4].

The roles of lipid-containing supra-structures, like extracellular vesicles, are revolutionizing concepts in cell biology and are also the foci of lipidomic scrutiny. Sagini and colls. analyzed the fatty acid profiling of extracellular vesicles released by senescent cells and found that they are selectively enriched in polyunsaturated and saturated chains, thus prompting intriguing hypotheses [7]. A similar concept, but arising from prokaryote cells, is that of outer membrane vesicles, which play a role in the pathogenic mechanisms of bacterial infections. Their lipidomic analysis has been addressed by Jasim and colls. in *Klebsiella pneumoniae*, providing valuable information towards unveiling the mechanisms of bacterial resistance to Polymyxin B [10].

Out of the pure lipidomic profiling, regulation of lipid metabolism is still an open field. Tian and colls. addressed the molecular mechanisms governing lipid accumulation in trophoblast cells via cell biology and molecular biology approaches [11]. The clinical side of sphingolipid metabolism has been addressed in the article by Malekkou and colls. in which the activity of non-lysosomal glucosylceramidase is evaluated in patients presenting mutations in the gene encoding the enzyme.

Novel synthetic lipids can modulate cell signaling. This is the case reported by Su and colls. who described the effects of ursodeoxycholyl lysophosphatidylethanolamide on integrin signaling and endocytic pathway [12].

The search for new biomaterials of therapeutic use is another goal of lipid-related studies. One example is the successful development of a matrix for the oral administration of hydrophobic compounds by Fratter and colls [3].

The six reviews included in this issue represent some of the main concerns of the community and can provide clues towards current needs as well as future directions. In all cases, the point towards biomedical topics. Public health issues, cancer, and cardiovascular disease are the foci of three of the articles. Two of these reviews address the role of polyunsaturated-derived mediators in hematologic malignancies [13], and the last review is related to the involvement of lipid metabolism modifications in the pathogenesis of viral infection-induced cancers [14]. Solati and colls. lecture on the impact of oxidative stress, in particular that of oxidized lipids on acute coronary disease [15]. The relevance of lipidomics analysis in glomerulopathies, a group of rare kidney diseases, the usefulness of lipid imaging, and the need for improved sensitivity and resolution is addressed by Abbas and colls [8]. A defective resolution of inflammation contributes to the pathogenesis of cystic fibrosis, another rare disease. Philippe and Urbach indicate the state of the art of lipid mediators of resolution in the context of this pathology [16]. Finally, Krivoi and Petrov review the functional role of cholesterol metabolism in neuromuscular junction, a key aspect to understanding the physiology of synaptogenesis and neural transmission, which has implications in motor disorders [17].

## 3. Future

Biomedical applications and clinical studies will benefit from analytical advances. Sensitivity improvements must be reflected by a subtler research, evolving from total cell to subcellular approaches. This may lead to the growth of “sublipidomics” as a branch of this discipline. Although the pathophysiology of human diseases represents the mainstream and the bulk of research on bioactive lipids and lipidomics, the search for new lipid base biomaterials and the roles and applications of natural substances should gain momentum in the following years. The main technical challenge is still the need to ensure accuracy in the identification of isomers, while the number of lipid molecules keeps expanding. Biomarker research and clinical lipidomics will be highly impacted by these developments. Also, MS imaging must progress in sensitivity and resolution to keep the pace and consolidate as a complementary approach to high resolution microscopy. Finally, studies integrating different omics disciplines are paving the path to a more global view and a better understanding of biological processes.

## Figures and Tables

**Figure 1:**
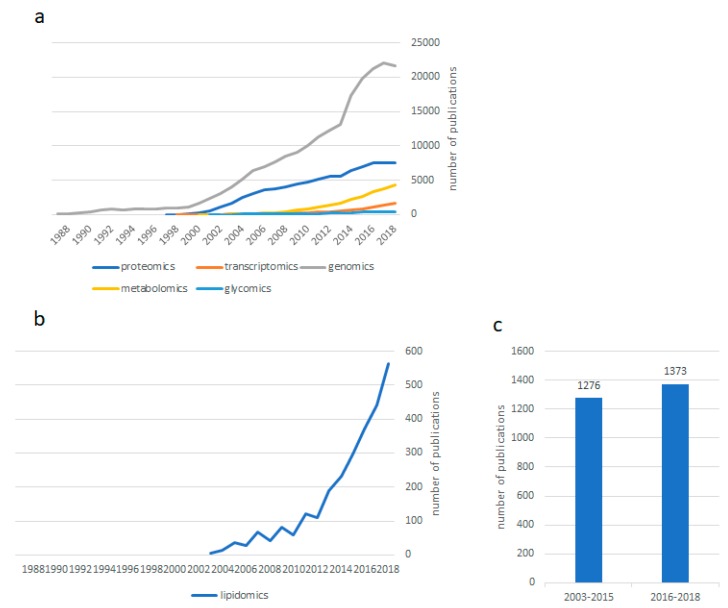
(**a**) Number of listed publications in PubMed over time using the corresponding “omic” discipline name as the keyword. (**b**) Number of listed publications in PubMed over time using “lipidomics” as the keyword. (**c**) Comparison of the number of listed publications in PubMed using “lipidomics” as the keyword during two periods of time, namely 2003–2015 and 2016–2018.
